# Exocomets in a planet nursery

**DOI:** 10.1038/s41467-026-78757-6

**Published:** 2026-10-12

**Authors:** Daniela Iglesias

**Affiliations:** 1 Astrophysics Research Cluster, School of Mathematical and Physical Sciences, The University of Sheffield, Sheffield, UK; 2 School of Physics & Astronomy, University of Leeds, Leeds, UK

**Keywords:** Exoplanets, Stars

## Abstract

Evidence for exocomets has been found in PDS 70, a young planetary system where planets are still forming. The discovery extends exocomet studies into an earlier stage of planetary evolution, when planets, planetesimals and protoplanetary discs coexist.

Exocomets are small icy bodies whose compositions and movements can provide clues to the formation and evolution of planetary systems. Their role in redistributing volatile material during planet formation, however, remains poorly understood. PDS 70 provides an unique opportunity to study how such bodies move through a forming planetary system. Its protoplanetary disc hosts at least two giant planets that have been directly imaged while still accreting material from the surrounding disc^1^. The planets have carved a large gap in the disc, separating the outer reservoir of gas and dust from the inner regions of the system (Fig. 1). Observations with the James Webb Space Telescope (JWST) have also revealed water vapour in the innermost disc, close to the star^2^. How that water arrived there remains unclear. It could have formed locally, or it could have been transported inward from more distant regions of the disc. Novais et al.^3^ report rapidly varying sodium absorption in PDS 70, a planetary system only about five million years old. They interpret these transient features as evidence for sublimating exocomets. If confirmed, the observations would provide a rare view of exocomets moving through a system while planets are still forming, offering new clues to how material is redistributed during the assembly of planetary systems.

**Fig. 1:**
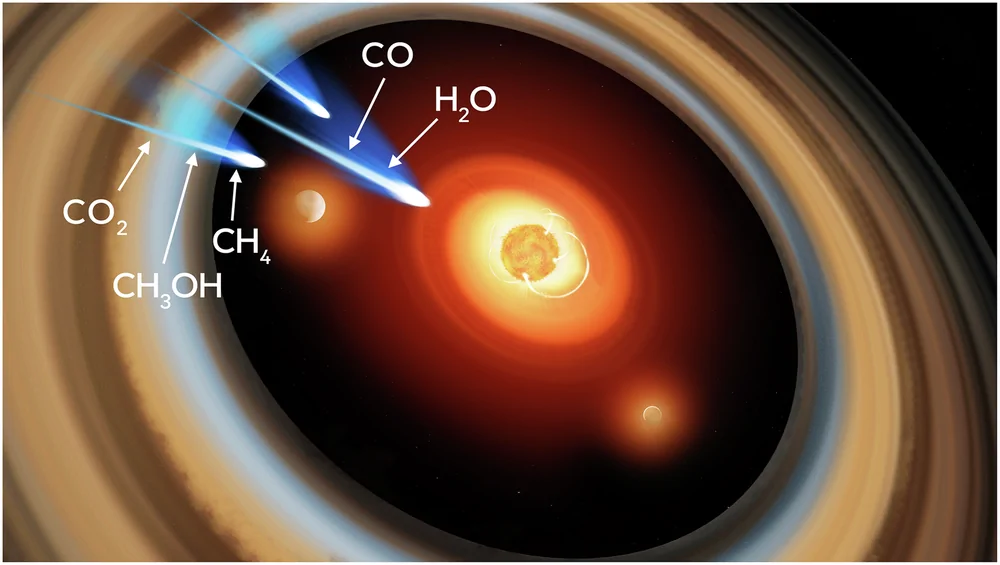
Artist’s impression of the young planetary system PDS 70, depicting exocomets transporting volatile material towards the inner system. As these icy bodies (white-blue features on the upper left) approach the star (center of the image), they could release molecules such as water (H_2_O), carbon monoxide (CO), carbon dioxide (CO_2_), methane (CH_4_), and methanol (CH_3_OH), potentially enriching the material available for forming planets. The giant protoplanets PDS 70 b and PDS 70 c are shown embedded within the disc cavity, illustrating the coexistence of planet formation and volatile transport in this young system. Illustration by Renaud Janin.

## The challenge of observing exocomets

Exocomets are difficult to observe directly. Unlike planets, they are too small to be imaged with current facilities. Their presence can instead be inferred from photometric and spectroscopic signatures. Photometric observations can reveal asymmetric or otherwise unusual transits caused by a cometary coma or tail passing in front of the star. Spectroscopically, gas released from a sublimating body can imprint transient absorption lines on the stellar spectrum. As a comet approaches its star, increasing irradiation can cause volatile material on its surface to sublimate, producing a cloud of gas that gives rise to narrow absorption features. Such events can last only days and are difficult to predict, making exocomets among the most elusive constituents of planetary systems.

The phenomenon is nevertheless well established in the young debris-disc system *β* Pictoris^4^. In that system, repeated transient absorption events have provided a window onto a population of bodies orbiting a planetary system at a later stage of its evolution. Their frequency, chemical signatures and changing velocities have revealed information about the cometary population and its dynamical environment^5^. Dynamical studies of the *β* Pictoris exocomets have even provided evidence for the gravitational influence of planets almost 20 years before those planets were directly detected^6–9^. Exocomets can therefore serve not only as remnants of planet formation, but also as tracers of the architecture and dynamical evolution of planetary systems.

## Exocomets in a planet-forming system

Novais et al.^3^ now report similar transient behaviour in PDS 70. Their analysis of archival spectra reveals rapidly varying sodium absorption lines that appear and disappear on timescales of days. The transient nature of the signals, together with their spectroscopic characteristics, resembles the absorption events long associated with exocomets in *β* Pictoris. Novais et al. argue that sublimating bodies provide the most plausible explanation for the observations. Further observations will be needed to establish the origin of the signals and to determine whether the inferred bodies are indeed exocomets.

The age of PDS 70 makes this result particularly significant. Most known exocomet systems are observed after the main stages of planet formation have ended^10^. Their cometary populations therefore offer information about the remnants and long-term dynamical evolution of planetary systems. PDS 70 presents a different opportunity. Here, giant planets are still accreting, the protoplanetary disc is evolving, and water vapour is already present in the inner system. Evidence for exocomet activity would therefore place minor bodies alongside planets and discs as active components of a planetary system still under construction.

The planetary architecture of PDS 70 could also be important for understanding how such bodies move through the system. Massive planets can strongly perturb the orbits of smaller bodies, scattering some onto trajectories that carry them towards the star while ejecting others from the system. In a young system containing a substantial outer reservoir of solids, such interactions could provide a mechanism for moving icy material across the large radial distances separating the cold outer disc from the warmer inner regions. The giant planets may therefore do more than sculpt the disc: they could also redistribute small bodies throughout the system.

## Exocomets as delivery vehicles

This raises the possibility that exocomets could contribute to the redistribution of volatile-rich material within young systems. In the Solar System, comets and other icy bodies are thought to have participated in the delivery of water and other volatile compounds, including carbon-bearing molecules, to the terrestrial-planet region^11–13^. If exocomets similarly transport organic material through young planetary systems, their redistribution could influence not only where volatiles accumulate, but also the chemical inventory available to newly forming planets. A young planetary system containing massive planets provides a natural environment in which icy bodies from outer regions could be dynamically perturbed and scattered towards the star. Such transport could link chemically distinct reservoirs during planet formation. Whether this is happening in PDS 70 remains an open question. The detection of transient sodium absorption by Novais et al.^3^ does not by itself demonstrate that the water vapour observed by JWST was delivered by the same population of bodies, nor does it establish how much volatile material exocomets might transport into the inner disc. The sodium signatures could instead trace a population of bodies whose primary significance is dynamical rather than chemical.

That possibility is itself important. Exocomets can provide information that is difficult to obtain from observations of planets alone because their orbits are readily perturbed by massive bodies. The distribution and timing of cometary activity could therefore encode information about the dynamical architecture of a planetary system. Applying this approach to a system as young as PDS 70 could therefore provide a new way of probing the dynamical interactions that accompany planet formation, including processes that may be difficult to observe directly. Distinguishing between these possibilities will require observations that identify additional events, constrain their velocities and orbital properties, and search for other chemical species associated with sublimating material.

## Exocomets as probes of planetary evolution

PDS 70 could mark a transition in exocomet research. For four decades, *β* Pictoris has been the principal laboratory for understanding these elusive bodies, providing insight into the remnants of planetary-system formation and their subsequent evolution. Evidence for exocomets in PDS 70 would extend this view into a much earlier phase, when planets, discs and minor bodies are evolving together. Rather than simply revealing what remains after planet formation, exocomets could provide a new way to trace how material moves through a planetary system as its architecture takes shape.

The next generation of time-domain and high-resolution spectroscopic observations should provide opportunities to test this picture. Detecting more transient events, determining their frequency and orbital properties, and searching for signatures of other volatile species will help establish how common exocomets are in young systems and what role they play in transporting material. A larger sample of young systems will also reveal whether PDS 70 is unusual or instead represents a common stage in planetary evolution. The key question is no longer simply whether comets exist around other stars, but what they can tell us about the systems in which they reside. PDS 70 may offer a glimpse of a previously inaccessible stage of exocomet evolution, in which the movements and compositions of small icy bodies reveal how planetary systems acquire their architecture and chemical inventory.

## References

[1] Haffert, S. Y. et al. Two accreting protoplanets around the young star PDS 70. *Nat. Astron.* **3**, 749–754 10.1038/s41550-019-0780-5 (2019).

[2] Perotti, G. et al. Water in the terrestrial planet-forming zone of the PDS 70 disk. *Nature* **620**, 516–520 10.1038/s41586-023-06317-9 (2023). PMC10432267 37488359

[3] Novais, A. et al. Potential sublimating exocomets around the young star PDS 70. *Nat. Commun.* **17**, 8894 10.1038/s41467-026-76880-y (2026). PMC13503679 42637760

[4] Ferlet, R., Hobbs, L. M. & Madjar, A. V. The beta Pictoris circumstellar disk. V. Time variations of the CA II-K line. *Astron. Astrophys.* **185** 267–270 (1987).

[5] Kiefer, F. et al. Two families of exocomets in the *β* Pictoris system. *Nature* **514**, 462–464 10.1038/nature13849 (2014). 25341784

[6] Beust, H., Lagrange-Henri, A. M., Vidal-Madjar, A. & Ferlet, R. The beta Pictoris circumstellar disk. X. Numerical simulations of infalling evaporating bodies. *Astron. Astraphys.* **236**, 202 (1990).

[7] Lagrange, A. M. et al. A probable giant planet imaged in the *β* Pictoris disk. VLT/NaCo deep L’-band imaging. *Astron. Astrophys.* **493**, L21–L25 10.1051/0004-6361:200811325 (2009).

[8] Lagrange, A. M. et al. Evidence for an additional planet in the *β* Pictoris system. *Nat. Astron.* **3**, 1135–1142, 10.1038/s41550-019-0857-1 (2019).

[9] Sutlieff, B. J. et al. Direct imaging discovery of giant exoplanet SS pictoris d: a decade-long game of hide-and-seek. * Astrophys. J. Lett.* **1006**, L10 10.3847/2041-8213/ae80a0 (2026).

[10] Iglesias, D. et al. An overview of exocomets. *Space Sci. Rev.* **221**, 122 10.1007/s11214-025-01247-6 (2025).

[11] Goesmann, F. et al. Organic compounds on comet 67P/Churyumov-Gerasimenko revealed by COSAC mass spectrometry. *Science* **349**, aab0689 10.1126/science.aab0689 (2015). 26228156

[12] Cordiner, M. A. et al. A D/H ratio consistent with Earth’s water in Halley-type comet 12P from ALMA HDO mapping. *Nat. Astron.* **9**, 1476–1485 10.1038/s41550-025-02614-7 (2025).

[13] Oba, Y. et al. Extraterrestrial hexamethylenetetramine in meteorites—a precursor of prebiotic chemistry in the inner solar system. *Nat. Commun.* **11**, 6243 10.1038/s41467-020-20038-x (2020). PMC7721876 33288754

